# Serotype Specific Primers and Gel-Based RT-PCR Assays for ‘Typing’ African Horse Sickness Virus: Identification of Strains from Africa

**DOI:** 10.1371/journal.pone.0025686

**Published:** 2011-10-20

**Authors:** Narender S. Maan, Sushila Maan, Kyriaki Nomikou, Manjunatha N. Belaganahalli, Katarzyna Bachanek-Bankowska, Peter P. C. Mertens

**Affiliations:** Vector-borne Disease Programme, Institute for Animal Health, Pirbright Laboratory, Pirbright, Woking, Surrey, United Kingdom; Royal Tropical Institute, Netherlands

## Abstract

African horse sickness is a devastating, transboundary animal disease, that is ‘listed’ by the Office International des Epizooties (OIE). Although attenuated, inactivated and subunit vaccines have been developed for African horse sickness virus (AHSV), these are serotype-specific and their effective deployment therefore relies on rapid and reliable identification of virus type. AHSV serotype is controlled by the specificity of interactions between neutralising antibodies, and components of the outer-capsid, particularly protein VP2 (encoded by AHSV genome segment 2 (Seg-2)). We report the development and evaluation of novel gel based reverse transcription-PCR (RT–PCR) assays targeting AHSV Seg-2, which can be used to very significantly increase the speed and reliability of detection and identification (compared to virus neutralisation tests) of the nine serotypes of AHSV. Primer sets were designed targeting regions of Seg-2 that are conserved between strains within each of the AHSV serotype (types 1 to 9). These assays were evaluated using multiple AHSV strains from the orbivirus reference collection at IAH (www.reoviridae.org/dsRNA_virus_proteins/ReoID/AHSV-isolates.htm). In each case the Seg-2 primers showed a high level of specificity and failed to cross-amplify the most closely related heterologous AHSV types, or other related orbiviruses (such as bluetongue virus (BTV), or equine encephalosis virus (EEV)). The assays are rapid and sensitive, and can be used to detect and type viral RNA in blood, tissue samples, or cultivated viral suspensions within 24 h. They were used to identify AHSV strains from recent outbreaks in sub-Saharan African countries. These methods also generate cDNAs suitable for sequencing and phylogenetic analyses of Seg-2, identifying distinct virus lineages within each virus-type and helping to identify strain movements/origins. The RT-PCR methods described here provide a robust and versatile tool for rapid and specific detection and identification of AHSV serotypes 1 to 9.

## Introduction

*African horse sickness virus* is a distinct virus species within the genus *Orbivirus* (of which *Bluetongue virus* is the ‘type species’), within the family *Reoviridae*. African horsesickness (AHS) is an important transboundary disease that is ‘listed’ by the Office International des Epizooties (OIE). It is an acute or sub-acute, non-contagious, arthropod-borne viral disease, characterized by severe pyrexia, widespread haemorrhages and oedematous exudations [1]. AHS has case fatality levels as high as 95% in horses and 80% in mules, while donkeys and zebras show few if any clinical signs and are thought to act as ‘reservoir’ hosts [2], [3]. The African horse sickness virus (AHSV) can also infect large carnivores via an oral route and may be a factor in the viability of endangered wildlife populations [4], [5].

AHS is endemic in sub-Saharan Africa [6] but single AHSV serotypes have occasionally expanded outside these boundaries, persisting in newly affected areas for a number of years [7]. In 1959–61 an outbreak caused by AHSV serotype 9 (AHSV-9) occurred in the Middle East, spreading as far as Pakistan and India, causing heavy mortality in equids [8]. In 1965, AHSV-9 caused an outbreak that started in North Africa but spread as far as southern Spain [7], [9]. In the late 1980s importation of a zebra infected with AHSV-4 from Namibia, caused an outbreak in Spain and Portugal [7], [10], [11], [12]. These incursions demonstrate that the geographic area suitable for successful transmission of AHSV is much greater than its current endemic zone and suggest that if AHS become fully established in Europe, it could inflict massive losses on the equine industry. Control measures have therefore been put in place by the European Community to regulate movements of equids [13] and combat this disease [14].

More recently (in 2007) AHSV-4 was identified in horses in Kenya, AHSV-2 and AHSV-7 in Senegal (ProMed Archive number: 20070915.3066, 20070911.3005, 20070825.2788, 20070627.2074, and 20070623.2031), Nigeria (ProMed Archive number: 20070308.0815, 20070131.0399), Ghana, Mali and Mauritania [7] and AHSV-9 was identified in Gambia in 2007, 2009 [15]. In 2008, AHSV-2 was also reported in Ethiopia (ProMed Archive number: 20080930.3087). There has subsequently been a major further increase in the number of AHSV types circulating in Ethiopia, with detection of AHSV serotypes 2, 4, 6, 8 and 9 during 2010 (Bankowska et al – in preparation).

Bluetongue virus (BTV) and AHSV are transmitted by similar *Culicoides* vector species [16]. Changes in the global distribution of BTV, with massive outbreaks caused by multiple BTV serotypes throughout Europe (since 1998), incursions into the south-eastern USA by previously exotic virus types, and detection of BTV-2 and 7 in Australia [17], [18] have been linked to a combination of increased international trade, recruitment of novel vector species, climate change and its effects on vector distribution [19], [20]. There have also been changes in the distribution of Epizootic haemorrhagic disease virus (EHDV), AHSV, and equine encephalosis virus (EEV) [21], suggesting that they could also emerge to pose important threats to European/global livestock and wildlife populations.

The AHSV genome consists of 10 segments of linear double-stranded RNA that encode seven structural proteins (VP1 to VP7) and four distinct non-structural proteins (NS1, NS2, NS3/NS3a and NS4) [22], [23], [24], [25], [26], [27]. The surface of the AHSV capsid is composed of VP2 (encoded by genome segment 2 (Seg-2)) and VP5 (encoded by Seg-6), which are primarily involved in cell attachment and penetration during initiation of infection. The specificity of reactions between these outer-capsid proteins (particularly VP2) and neutralising antibodies generated by the mammalian host can be used to identify and distinguish nine AHSV serotypes in serum neutralisation tests (SNT) or virus neutralisation tests (VNT) [28], [29], .

Based on field observations, these different AHSV serotypes have previously been linked to differences in immunogenicity, production of different disease patterns and virulence [35], [36]. However, the multi-segmented nature of the AHSV genome and the potential for field strains to efficiently exchange/reassort genome segments, suggest that these characteristics may not be solely due those genome segments or proteins that control serotype [37]. Recent experiences with the control of BTV-1 and 8 in northern Europe indicate that vaccination of horses against the AHSV serotype responsible for an outbreak would provide an effective control measure. However, rapid deployment of an appropriate vaccine strain depends on identification of the serotype involved, as quickly and accurately as possible.

Conventional serotyping methods rely on AHSV isolation from clinical specimens, in suckling mouse brain and adaptation to tissue culture (BHK-21, Vero or KC (*Culicoides soronensis*) cells), followed by serological assays (VNT), taking a minimum of 3–4 weeks. Alternatively it may be possible to identify the virus serotype by the reaction of antisera from an infected animal with reference strains of each AHSV serotype. However, these methods require access to stocks of well characterised reference antisera or reference virus strains for each serotype and cannot distinguish individual virus strains within a single orbivirus serotype. Consequently they are unsuitable for high precision ‘molecular’ epidemiological studies to differentiate closely related virus lineages, or topotypes. They can also be complicated by co-circulation of different viruses, leading to the generation of a broadly cross-reactive neutralising-antibody responses after sequential infection with more than one serotype [38]. The resulting delays in vaccine deployment could allow the early spread and consequently the duration of an epizootic.

Serotype-specific RT-PCR assays have been developed for the 26 serotypes of BTV [39], [40], [41]. Attempts to design similar primers and typing assays targeting Seg-2 of AHSV [42] have previously been hampered by a lack of full length Seg-2 sequence data for all AHSV serotypes. Generation of sequence data for Seg-2 from multiple reference and field strains of the nine serotypes of AHSV [31], [43, Bankowska et al – manuscript in preparation] has clarified the level and extent of sequence variation within and between different serotypes.

We describe the design and *in silico* evaluation of Seg-2 based oligonucleotide primers and RT-PCR assays for each AHSV serotype. Assay specificity was evaluated with a range of AHSV isolates from dsRNA virus reference collection at IAH [44]. They were also used to identify AHSV strains circulating in East and West Africa, using RNA samples extracted directly from diagnostic blood or tissue samples. These techniques remove the need for virus isolation and ‘reference antisera’, increasing the speed and availability of virus identification methods, and reducing the use of experimental animals.

## Materials and Methods

### AHSV reference and field isolates

Different AHSV isolates (listed in Table 1) from the Institute for Animal Health (IAH) ‘Orbivirus Reference Collection’ (ORC) [44] were grown in BHK-21 clone 13 (European Collection of Animal cell Cultures (ECACC – 84100501))/Vero (ECACC – 84113001) cell monolayers, until 40–100% cytopathic effect (CPE) was observed and in *C. sonorensis* (KC) cells (originally provided by colleagues at the USDA lab in Laramie, Wyoming) for 7 days. The field samples used in these studies were taken from naturally infected animals in the field, by qualified veterinarians, as part of normal veterinary care and diagnostic testing procedures in the respective countries. No samples were taken from animals specifically for these studies, so we believe that ethical approval is not required.

**Table 1:** List of AHSV isolates used in this study.

Virus serotype^1^	Origin	IAH-P^3^ Reference collection number	Passage history (MB^4^/BHK^5^) (V^6^/KC1^7^/?^8^)	Other details regarding samples
**AHSV Reference Strains**				
**AHSV1**	RSA^2^	RSArah1/03	MB3/BHK10	
**AHSV2**	RSA	RSArah2/03	MB3/BHK7	
**AHSV3**	RSA	RSArah3/03	?/BHK2	
**AHSV4**	RSA	RSArah4/03	?/BHK2	
**AHSV5**	RSA	RSArah5/03	MB3/BHK7	
**AHSV6**	RSA	RSArah6/03	MB3/BHK6	
**AHSV7**	RSA	KENrah7/03	MB4/BHK9	
**AHSV8**	RSA	RSArah8/03	?/BHK2	
**AHSV9**	RSA	PAKrah9/03	?/BHK2	
**AHSV Field Strains**				
**AHSV2**	Ethiopia	ETH2010/01	KC1	
**AHSV2**	Senegal	SEN2007/01	MB1/BHK2	Sent from ‘The Republique du Senegal, Ministere de l'elevage’ in May 2007
**AHSV2**	Senegal	SEN2007/02	MB1/BHK3	Same as SEN2007/01 (see above)
**AHSV2**	Senegal	SEN2007/03	MB1/BHK3	From spleen of a six year old male horse, sampled on 26th August 2007 in Diourbel, Senegal.
**AHSV2**	Senegal	SEN2007/04	MB1/BHK3	From lung of a three year old female horse, sampled on 2nd September 2007 from Lerane Sambou village, Senegal.
**AHSV2**	Senegal	SEN2007/05	MB1/BHK3	From spleen of a six year old male horse, sampled on 24th August 2007 from Diongo village, Diourbel, Senegal.
**AHSV4**	Kenya	KEN2007/01	V3	From a horse sampled in Kenya during October 2007 and passed on Vero cell culture at the Central Veterinary Research Laboratory (CVRL), Dubai.
**AHSV4**	Kenya	KEN2007/02	V2/KC1	Same as KEN2007/01 (see above)
**AHSV4**	Spain	SPA1987/01	MB1/BHK3	
**AHSV6**	Ethiopia	ETH2010/19	KC1	
**AHSV7**	Senegal	SEN2007/06	V3	From lung sample of a male horse from Guede Bousso village Senegal in September 2007
**AHSV7**	Ethiopia	ETH2010/09	V1	
**AHSV8**	Ethiopia	ETH2010/10	KC1	
**AHSV8**	Ethiopia	ETH2010/11	KC1	
**AHSV9 & 2**	Ethiopia	ETH2010/23	KC1	
**AHSV9 vaccine**	Senegal	SENvvvv/09	?/BHK2	The Senegal AHSV-9 monovalent vaccine was identified as ‘an original vaccine lot M_0107, BEST BEFORE FEB 2009’

1 Bankowska et al – in preparation;

2 RSA: Republic of South Africa;

3 Institute for Animal Health, Pirbright.

4 Number of passages in mouse brain;

5 Number of passages in BHK-21 cells;

6 Number of passages in Vero cells.

7 Number of passages in KC (*Culicoides soronensis*) cells;

8 Unknown.

IAH-P ‘dsRNA virus reference collection number’ composed of ‘country code, year, and the number of the isolate in that year from that country. Full details of these isolates are available on the dsRNA virus reference collection website [44].

### Extraction of Viral dsRNA

RNA was extracted from BTV infected cell pellets using Trizol® (Invitrogen) [45], or from cell-free tissue culture supernatant using the QIAamp Viral RNA Mini Kit (Qiagen), or a Universal BioRobot (Qiagen), according to manufacturer's instructions.

### Group specific RT-PCR assay

RNA samples extracted from clinical samples or cell culture material, were tested in duplicate by conventional RT-PCR assays using AHSV genome segment 7 (Seg-7) specific primers [46]. Samples that generated a strong cDNA band of the expected size (1179 Kb), were considered to contain AHSV RNA and were used for further processing/testing.

### Serotype specific RT-PCR assays

Multiple primer-pairs were designed based on sequence data for Seg-2, targeting each of the nine AHSV serotypes (Table S1). cDNA copies were reverse transcribed from denatured dsRNA templates, then amplified by One-Step RT-PCR kit (Qiagen) as described by Mertens et al. [41], or by a one-step RT-PCR system (Invitrogen) and high fidelity platinum® Taq as described by Maan et al [47]. In some cases, full length cDNA copies of AHSV Seg-2 were also synthesised and sequenced, as described by Maan et al [48]. RT-PCR typing results were also confirmed by sequencing of amplified cDNA products from Seg-2 and sequence data analysed using MAFFT [49] and/or Clustal W programs [50]. Phylogenetic comparisons were made using Neighbour-Joining (NJ) algorithms and the trees were constructed in Mega 4.1 [51] using nucleotide sequences in a pair-wise deletion, p-distance algorithm, and bootstrapped using 500 replicates.

## Results

### Development of RT-PCR assays and their evaluation for type specificity

Comparisons of full-length Seg-2 sequences for reference strains of the nine AHSV serotypes and other field isolates [31], [43], [52], [53, Bankowska et al – in preparation], were used to identify regions of conservation within individual AHSV serotypes and variability between different types. These regions were used to design a minimum of two serotype-specific primer-pairs for use in RT-PCR assays to detect each AHSV serotype (Table S1).

**Figure 1:**
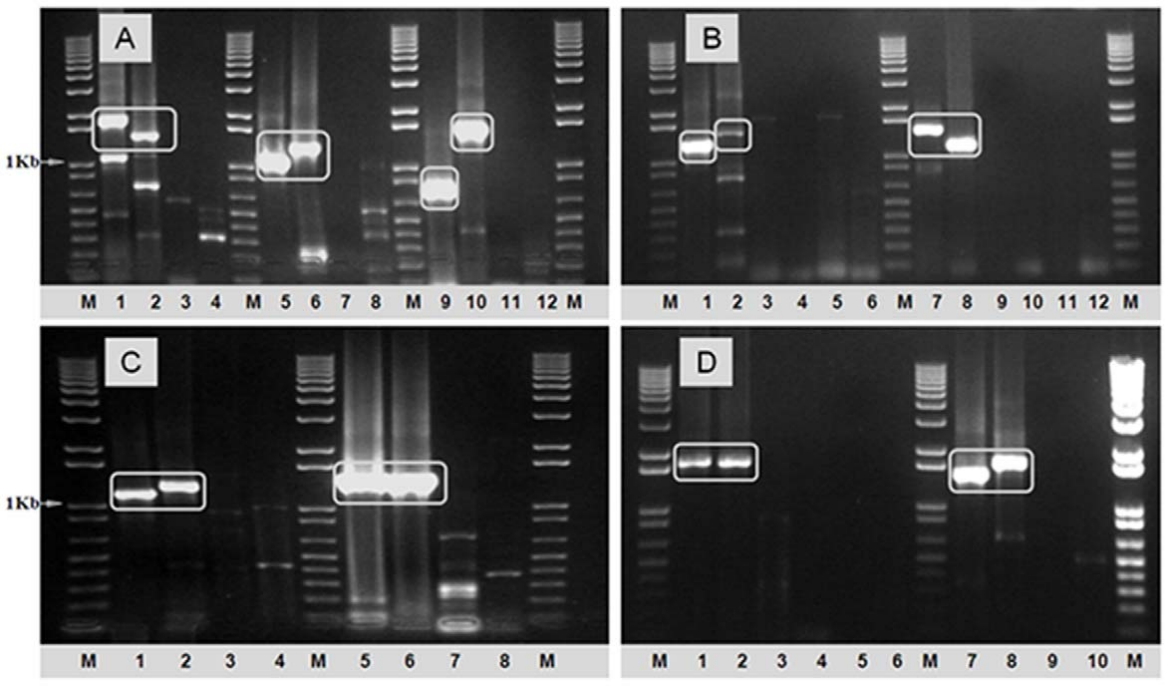
‘Type-specific’ primers for Seg-2 of AHSV-1 to AHSV-9. The cDNAs amplified from Seg-2 of AHSV 1–9 serotypes using type-specific primers were analyzed by 1% agarose-gel-electrophoresis (AGE). **Panel A:** Lanes 1 and 2 show products from AHSV-1/RSArah1/03 using primer-pairs ‘1A1’ (1793 bp) and ‘1A2’ (1452 bp) respectively. Lanes 3 and 4: Primer-pairs ‘1A1’ and ‘1A2’ amplified only small amounts of ‘incorrectly’ sized products from AHSV-2/RSArah2/03 (heterologous-control). Lanes 5 and 6 show products from AHSV-2/RSArah2/03 using primer-pairs ‘2A1’ (1098 bp) and ‘2A2’ (1339 bp) respectively. Lanes 7 and 8: Primer-pairs ‘2A1’ amplified none but ‘2A2’ amplified only small amounts of ‘incorrectly’ sized products from AHSV-1/RSArah1/03 (heterologous-control). Lanes 9 and 10 show products from AHSV-3/RSArah3/03 using primer-pairs ‘3A1’ (751 bp) and ‘3A2’ (1524 bp) respectively. Lanes 11 and 12: Primer-pairs ‘3A1’ and ‘3A2’ showed no amplification from AHSV-7/KENrah7/03 (heterologous-control). **Panel B:** Lanes 1 and 2 show products from AHSV-4/RSArah4/03 using primer-pairs ‘4A1’ (1264 bp) and ‘4A3’ (1471 bp) respectively. Lanes 3, 4, 5 and 6: Primer-pairs ‘4A1’ and ‘4A3’ did not amplify Seg-2 from AHSV-5/RSArah5/03 and AHSV-8/RSArah8/03 respectively (heterologous-control). Lanes 7 and 8 show products from AHSV-5/RSArah8/03 using primer-pairs ‘5A2’ (1423 bp) and ‘5A3’ (1139 bp) respectively. Lanes 9, 10, 11 and 12: Primer-pairs ‘5A2’ and ‘5A3’ did not amplify Seg-2 from AHSV-4/RSArah4/03 and AHSV-8/RSArah8/03 respectively (heterologous-control). **Panel C:** Lanes 1 and 2 show products from AHSV-6/RSArah6/03 using primer-pairs ‘6A1’ (1154 bp) and ‘6A2’ (1270 bp) respectively. Lanes 3 and 4: Primer-pairs ‘6A1’ and ‘6A2’ amplified only small amounts of ‘incorrectly’ sized products from AHSV-8/RSArah8/03 (heterologous-control). Lanes 5 and 6 show products from AHSV-7/KENrah7/03 using primer-pairs ‘7A1’ (1426 bp) and ‘7A2’ (1426 bp) respectively. Lanes 7 and 8: Primer-pairs ‘7A1’ and ‘2A2’ amplified only small amounts of ‘incorrectly’ sized products from AHSV-3/RSArah3/03 (heterologous-control). **Panel D:** Lanes 1 and 2 show products from AHSV-8/RSArah3/03 using primer-pairs ‘8A1’ (1757 bp) and ‘8A2’ (1732 bp) respectively. Lanes 3, 4, 5 and 6: Primer-pairs ‘8A1’ and ‘8A2’ did not amplify Seg-2 from AHSV-4/RSArah4/03 and AHSV-5/RSArah5/03 respectively (heterologous-control). Lanes 7 and 8 show products from AHSV-9/PAKrah9/03 using primer-pairs ‘9A1’ (1483 bp) and ‘9A2’ (1706 bp) respectively. Lanes 9 and 10: Primer-pairs ‘9A1’ and ‘9A2’ did not amplify Seg-2 from AHSV-6/RSArah6/03 (heterologous-control). Lane M: 1 Kb DNA marker (Invitrogen).

The RT-PCR primers were tested in reactions containing RNA extracted from BHK-21 cells infected with the different AHSV serotypes (Table 1). The cDNAs generated, were analysed by 1% agarose gel electrophoresis (AGE), to check both their size and relative level of synthesis. In each case cDNAs of the expected size were generated from RNA of the homologous serotype (Figure 1 panels A–D). None of the primer-pairs amplified cDNAs from RNA of even the most closely related heterologous AHSV types, or from members of other related *Orbivirus* species (data not shown). These initial evaluations confirm *in silico* analyses, indicating that the primers listed in Table S1 are indeed ‘type-specific’.

#### Identification of AHSV-2 isolates from Senegal 2007

**Figure 2:**
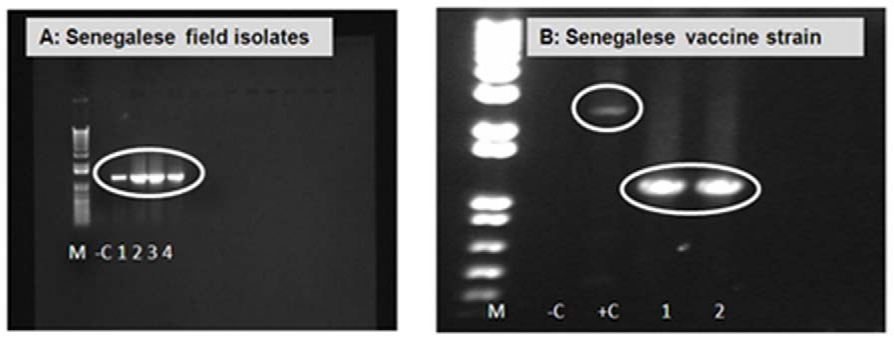
Electrophoretic analysis of cDNA products generated in AHSV group-specific RT-PCR assays using Seg-7 specific primers. PCR amplicons of 1179 bp were generated from AHSV Seg-7 specific RT-PCR of dsRNA extracted from equine lung and spleen extracts and vaccine strain from Senegal tested in duplicate (SEN2007/01 – lanes 1 and 2; SEN2007/02 – lanes 3 and 4 in panel A; SENvvvv/09 – lanes 1 and 2 in panel B respectively) [46], demonstrating that the samples contain AHSV RNA. Lane −C is a negative water control showing no amplification. Lane M: 1 Kb marker (Invitrogen). RNA from BTV-4/RSArrrr/04 was used as a positive control using primer-pair ‘4W2’ - 2324 bp [41] (lane +C, panel B).

**Figure 3:**
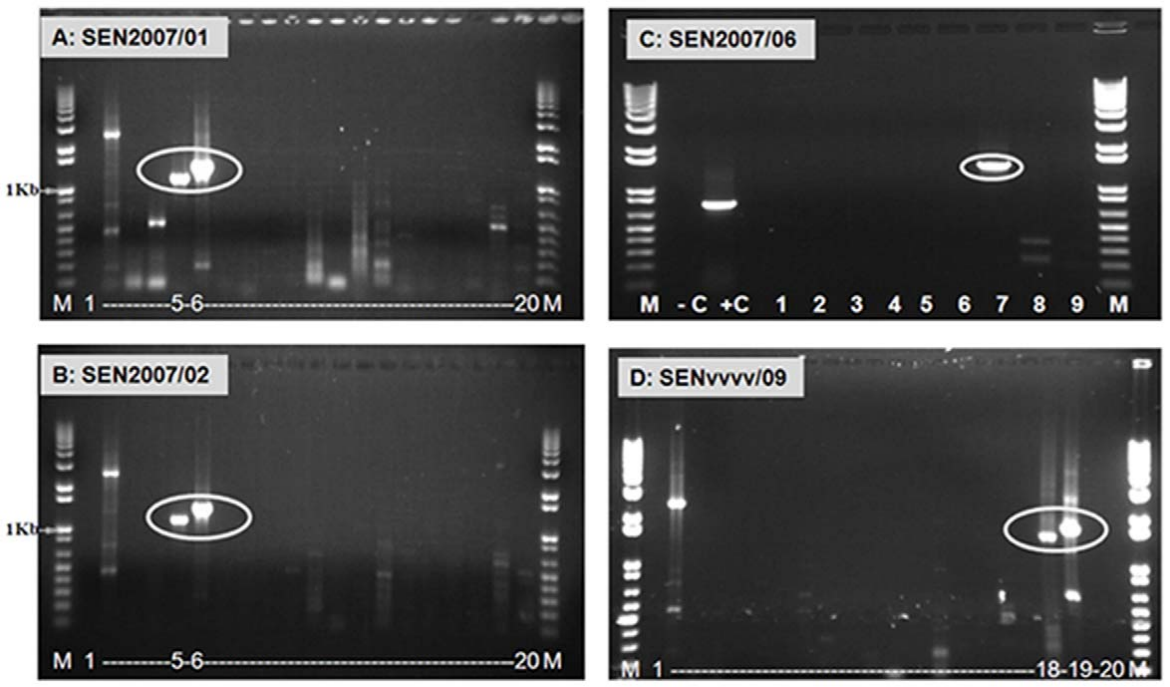
Electrophoretic analysis of cDNA from Seg-2 of AHSV isolates from Senegal 2007 using ‘type-specific’ primer-pairs. Panel A and B: PCR amplicons were generated from AHSV Seg-2 specific RT-PCR of dsRNA extracted from equine lung (SEN2007/01 – panel A) and spleen (SEN2007/02 – panel B) using primer-pairs ‘2A1’ (1098 bp, lane 5 in both panels) and ‘2A2’ (1339 bp, lane 6 in both panels) (Table S1), demonstrating that the samples contain AHSV-2 RNA. Lanes 3–20 represent RNA tested from AHSV-1 to 9 using two pairs of primers for each serotypes. Lane 1 is a negative water control showing no amplification. Lane M: 1 Kb marker (Invitrogen). RNA from BTV-4/RSArrrr/04 was used as a positive control using primer-pair ‘4W2’ - 2324 bp [41] (lane 2). **Panel C:** PCR amplicons were generated from AHSV Seg-2 specific RT-PCR of dsRNA extracted from SEN2007/06 using primer-pairs ‘7A1’ (1426 bp - lane 7) (Table S1), demonstrating that the samples contain AHSV-7 RNA. Lanes 1–9 represent RNA tested from AHSV-1 to 9 using first set of primers for each serotypes. No amplification was detected in other serotypes. Lane −C is a negative water control showing no amplification. Lane M: 1 Kb marker (Invitrogen). RNA from ASHV multivalent vaccine strain (SENvvv1/MV) with ‘3A1’ was used as positive control which generated a product of 751 bp (lane +C). **Panel D:** PCR amplicons were generated from AHSV Seg-2 specific RT-PCR of dsRNA extracted from SENvvvv/09 using primer-pairs ‘9A1’ (1483 bp - lane 18) and ‘9A2’ (1706 bp – lane 19) (Table S1), demonstrating that this sample contains AHSV-9 RNA. Lanes 3–20 represent RNA tested from AHSV-1 to 9 using two pairs of primers for each serotypes. Lane 1 is a negative water control showing no amplification. Lane M: 1 Kb marker (Invitrogen). RNA from BTV-4/RSArrrr/04 was used as a positive control using primer-pair ‘4W2’ - 2324 bp [41] (lane 2).

**Figure 4:**
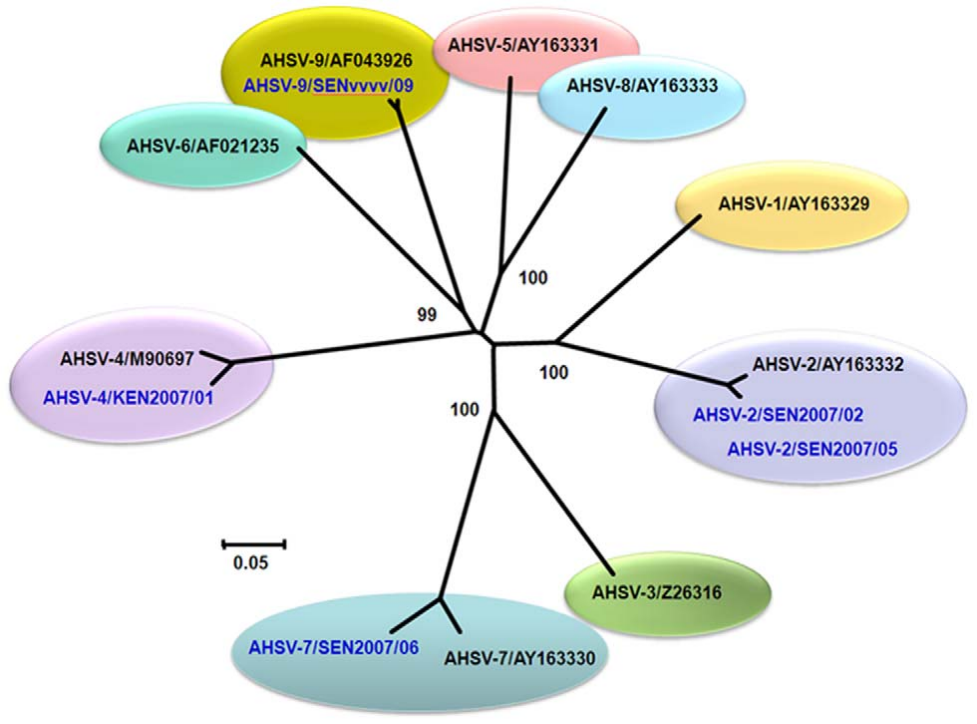
Neighbour-Joining tree, showing relationships in Seg-2 between reference and field strains of AHSV. The tree was constructed using distance matrices, generated using the p-distance determination algorithm in MEGA 4.1 (500 bootstrap replicates) [51]. AHSV split into distinct groups based on Seg-2 sequences, reflecting serological relationships between virus strains [31]. Reference strains of AHSV are shown in black and field isolates are shown in blue font. Isolate designations: IAH ‘dsRNA virus reference collection number composed of ‘country code, year, and the number of the isolate in that year from that country [44]. Seg-2 sequences of two Senegalese AHSV-2 field 2007 strains showed 99.8% nucleotide (nt) identity to each other and showed 97% nt identity to the AHSV-2 reference strain (500–1648 nt). AHSV-4/KEN2007/01 showed 96% nucleotide identity to the AHSV-4 published Seq-2 sequence in the upstream 1170 base pairs (480–1650 nt). Seg-2 (550–1800 nt) of Senegalese AHSV-7 strain from 2007 showed 92% identity to the AHSV-7 South African reference strain. Seg-2 (520–1684 nt) of Senegalese vaccine strain of type 9 showed 98.8% identity to the AHSV-9 South African reference strain. Scale represents number of substitutions per site. Values at major branching points represent NJ bootstraps.

RNA was extracted from equine samples, or from cell culture supernatants infected with these viruses (SEN2007/01, SEN2007/02, SEN2007/03, SEN2007/04 and SEN2007/05). In each case a strong +ve band of the expected size (1179 Kb) was generated in Seg-7 group-specific RT-PCR assays (Figure 2 – panel A). The RNA samples were also tested by conventional RT-PCR, using Seg-2 specific primers listed in Table S1. Strong +ve bands of the expected sizes were only generated with primer-pairs ‘2A1’ and ‘2A2’, demonstrating the presence of AHSV-2 RNA. None of the other AHSV types were detected, including AHSV-9 (which had recently been used to vaccinate the animals) (Figure 3). The AHSV-2 Seg-2 amplicons were sequenced, generating data for nucleotides (nt) 500–1648, which showed 97% nt identity with Seg-2 of the South African reference strain of AHSV-2 (Ac. No. AY163332) (Figure 4).

#### Identification of AHSV-7 from Senegal 2007

AHSV isolate (SEN2007/06) derived from a horse that died in 2007, was also tested in type-specific RT-PCR assays for each of the nine AHSV serotypes. Assays containing only one of the type 7 specific primers (‘7A1’) (Table S1) generated a band of the expected size (1426 bp) indicating that this sample contains AHSV-7 (Figure 3 – panel C). Although the other type 7 specific primers (‘7A2’) worked well with the reference strain RSArah7/03 and Ethiopian strain (ETH2010/09), they failed to amplify sequences from SEN2007/06, suggesting a significant variation in the footprint sequence for this primer-pair compared to the reference strain (Table S1). The ‘7A1’ Seg-2 amplicon (nt 550–1800) from SEN2007/06, was also sequenced, showing 92% nt identity overall to Seg-2 of the South African AHSV-7 reference strain (Ac. No. AY163330) confirming the isolate as AHSV-7 (Figure 4).

#### AHSV-9 vaccine strain from Senegal 2007

The virus species of the original Senegal vaccine strain (SENvvvv/09) was confirmed (as AHSV) by the synthesis of an 1179 Kb cDNA in group-specific RT-PCR assays containing Seg-7 primers (Figure 2). In subsequent type-specific assays, only two primer sets targeting Seg-2 of AHSV-9 (‘9A1’ and ‘9A2’) (Table S1) generated strong cDNA bands of the expected size, indicating that the vaccine did not contain other AHSV types (Figure 3 – panel D). The Seg-2 amplicons were sequenced (nt 520–1684) showing 98.8% nt identity with Seg-2 of the South African AHSV-9 reference strain (Ac. No. AF043926) confirming the virus type (Figure 4).

#### AHSV-4 isolates from Kenya 2007

**Figure 5:**
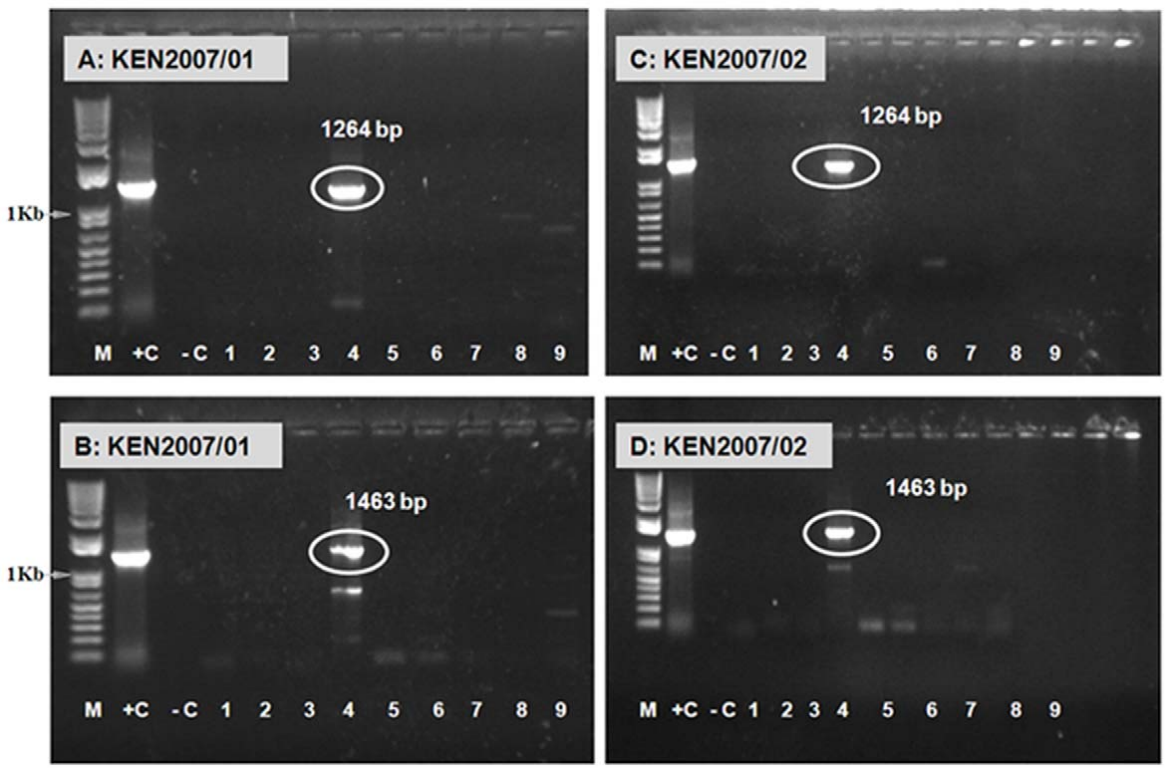
Electrophoretic analysis of cDNA from Seg-2 of AHSV isolates from Kenya 2007 using ‘type-specific’ primer-pairs. PCR amplicons were generated from AHSV Seg-2 specific RT-PCR of dsRNA extracted from KEN2007/01 and KEN2007/02 using primer-pairs ‘4A1’ (1264 bp, lane 4 in panels A and C) and ‘4A2’ (1463 bp, lane 4 in panels B and D) (Table S1), demonstrating that the samples contain AHSV-4 RNA. Lanes 1–9 represent RNA tested from AHSV-1 to 9 using pair of primers for each serotypes. Lane −C is a negative water control showing no amplification. Lane M: 1 Kb marker (Invitrogen). RNA from ASHV-2 from Senegal (SEN2007/02) with primer set ‘2A2’ (generating a product of 1339 bp) was used as a positive control.

RNA extracted from Kenyan isolates KEN2007/01 and KEN2007/02 were tested in conventional type-specific RT-PCR assays targeting Seg-2 (Figures 4 and 5). Only primer-pairs ‘4A1’, ‘4A2’ and ‘4A3’ generated strong bands of the expected sizes (1264 bp, 1463 bp and 1471 bp respectively) identifying the virus as AHSV-4. This indicates that these isolates did not contain RNA from other recognised AHSV serotypes. The Seg-2 amplicons were sequenced, showing 96% nt identity with Seg-2 of the South African AHSV-4 reference strain (Ac. No. M90697) confirming the virus type (Figure 4).

## Discussion

AHSV Seg-2 encodes VP2 (the main serotype-specific antigen and most variable of the virus proteins) and shows 47.6% to 71.4% nt identity between types, which can therefore be identified by phylogenetic comparisons [31], [43, Bankowska et al – in preparation]. Cross-hybridization methods [54] or hybridization and real-time RT-PCR methods targeting AHSV Seg-2 have previously been described [55], [56]. Sailleau, et al [42] have also reported typing of AHSV using a serogroup-specific forward-primer and a serotype-specific reverse-primer, based on a limited Seg-2 dataset. These primer-pairs generated a small amplicon for each of nine serotypes of AHSV and were used to ‘type’ a limited number of reference and field strains. The RT-PCR assay results obtained were also compared with VNT results but were not confirmed by sequencing of the resulting amplicons.

Recent full length sequencing of Seg-2 from reference strains and multiple field strains of AHSV (Bankowska et al – in preparation), have supported the design of additional AHSV ‘type-specific’ primer-pairs. We have successfully evaluated these primers, RT-PCR assays, sequencing and phylogenetic analyses, for differentiation of individual AHSV strains even within a single serotype. These techniques can determine AHSV serotype with greater accuracy, sensitivity and significantly more quickly than is possible by current serological techniques, facilitating surveillance and molecular epidemiology studies of the virus.

The relatively small number of AHSV isolates (N = 26 (Table 1); N = 107 *in silico* (Table S1)) that are available for sequence analyses and testing, has limited wider validation of the assays developed reported here. The specificity of the primers therefore primarily reflects their failure to recognise and amplify Seg-2 of other AHSV serotypes, rather than an ability to detect all possible isolates of the homologous type. If in future studies, any of these primers fail to detect Seg-2 of a novel isolate, it will be sequenced, providing a basis for further ‘evolution’ of primer sequences and assays. The ‘fine-scale’ differentiation and identification of individual AHSV strains, depends on the availability and characterisation of well documented reference isolates from different locations and belonging to different virus lineages. These need to be stored and maintained in a suitable reference collection (like the ORC) with links to relevant sequence data [44].

Recent disease outbreaks caused by AHSV-4 in Kenya (in 2007); AHSV-2, AHSV-7 and AHSV-9 in Senegal (in 2007), were identified using the ‘type-specific’ assays (as described here). Serotype 9 was also identified in the Gambia (in 2009) along with multiple serotypes in Ethiopia (in 2010) using the same techniques [15], Bankowska et al, in preparation]. These novel laboratory tools and the standardised protocol are easy to follow and do not require reference antisera or virus isolates that may have limited availability and which themselves represent a potential disease security risk. These methods are robust and versatile, reliably identifying AHSV type within 24 h of sample receipt, with no evidence of cross-amplification of other related types or other orbiviruses. Although slower than real-time RT-PCR assays, these conventional type-specific primers and assays can be used to generate cDNA amplicons for sequence analyses, providing definitive proof of relationships between Seg-2 of individual virus strains.

These phylogenetic comparisons will help to determine the prevalence, distribution and movements of different AHSV serotypes and topotypes, improving our understanding of the epidemiology and transmission of the virus, providing a basis for the design and rapid implementation of control strategies, including vaccination.

## Supporting Information

Table S1 **Primers for specific amplification of Seg-2 from various AHSV serotypes in RT-PCR assays.**
(DOC)

## References

[1] Stoltz MA, van der Merwe CF, Coetzee J, Huismans H (1996). Subcellular localization of the nonstructural protein NS3 of African horsesickness virus.. Onderstepoort J Vet Res.

[2] Hess WR (1988). The Arbovirus: Epidemiology and Ecology.

[3] OIE (2000). Manual of Standards for Diagnostic Tests and Vaccines.

[4] Alexander KA, Kat PW, House J, House C, O'Brien SJ (1995). African horse sickness and African carnivores.. Vet Microbiol.

[5] Van Rensberg IB, De Clerk J, Groenewald HB, Botha WS (1981). An outbreak of African horsesickness in dogs.. J S Afr Vet Assoc.

[6] Coetzer JAW, Guthrie AJ (2004). African horse sickness. 2nd ed.

[7] Wilson A, Mellor PS, Szmaragd C, Mertens PP (2009). Adaptive strategies of African horse sickness virus to facilitate vector transmission.. Vet Res.

[8] Mellor PS, Mertens PPC, Mahy BWJ, van Regenmortel MHW (2008). African horse sickness.. Encyclopedia of Virology. 3rd ed.

[9] Mellor PS, Hamblin C (2004). African horse sickness.. Vet Res.

[10] Mellor PS (1993). African horse sickness: transmission and epidemiology.. Vet Res.

[11] Rodriguez-Sanchez B, Fernandez-Pinero J, Sailleau C, Zientara S, Belak S (2008). Novel gel-based and real-time PCR assays for the improved detection of African horse sickness virus.. J Virol Methods.

[12] Lubroth J (1988). African horse sickness and the epizootic in Spain 1987.. Equine Practice.

[13] EEC (1990).

[14] EEC (1992).

[15] Oura CAL, Ivens PAS, Bachanek-Bankowska K, Bin-Tarif A, Jallow DB (2011). African Horse Sickness in The Gambia: Circulation of a live attenuated vaccine-derived strain.. Epidemiology and Infection.

[16] Du Toit RM (1944). The transmission of bluetongue and horse-sickness by Culicoides.. Onderstepoort J Vet Sci Anim Ind.

[17] NAMP (2009). The National Arbovirus Monitoring Program (NAMP) report 2008–2009.. http://www.animalhealthaustralia.com.au/wp-content/uploads/2011/03/nampar_0809.pdf.

[18] OIE (2008). Report Date: 11/09/2008, Country: Australia.. http://web.oie.int/wahis/public.php?page=report_ann_sem&country=AUS&year=2008&semester=1&aquatic=2&WAHID=1.

[19] Purse BV, Mellor PS, Rogers DJ, Samuel AR, Mertens PP (2005). Climate change and the recent emergence of bluetongue in Europe.. Nat Rev Microbiol.

[20] Purse BV, Brown HE, Harrup L, Mertens PP, Rogers DJ (2008). Invasion of bluetongue and other orbivirus infections into Europe: the role of biological and climatic processes.. Rev Sci Tech.

[21] Maclachlan NJ, Guthrie AJ (2010). Re-emergence of bluetongue, African horse sickness, and other orbivirus diseases.. Vet Res.

[22] Belhouchet M, Mohd Jaafar F, Tesh R, Grimes J, Maan S (2010). Complete sequence of Great Island virus and comparison with the T2 and outer-capsid proteins of Kemerovo, Lipovnik and Tribec viruses (genus Orbivirus, family Reoviridae).. J Gen Virol.

[23] Roy P, Mertens PP, Casal I (1994). African horse sickness virus structure.. Comp Immunol Microbiol Infect Dis.

[24] Firth AE (2008). Bioinformatic analysis suggests that the Orbivirus VP6 cistron encodes an overlapping gene.. Virol J.

[25] Diprose JM, Burroughs JN, Sutton GC, Goldsmith A, Gouet P (2001). Translocation portals for the substrates and products of a viral transcription complex: the bluetongue virus core.. EMBO J.

[26] Gouet P, Diprose JM, Grimes JM, Malby R, Burroughs JN (1999). The highly ordered double-stranded RNA genome of bluetongue virus revealed by crystallography.. Cell.

[27] Grimes JM, Burroughs JN, Gouet P, Diprose JM, Malby R (1998). The atomic structure of the bluetongue virus core.. Nature.

[28] Van Dijk AA, Huismans H (1982). The effect of temperature on the in vitro transcriptase reaction of bluetongue virus, epizootic haemorrhagic disease virus and African horsesickness virus.. Onderstepoort J Vet Res.

[29] Burrage TG, Trevejo R, Stone-Marschat M, Laegreid WW (1993). Neutralizing epitopes of African horsesickness virus serotype 4 are located on VP2.. Virology.

[30] Castillo-Olivares J, Calvo-Pinilla E, Casanova I, Bachanek-Bankowska K, Chiam R (2011). A modified vaccinia Ankara virus (MVA) vaccine expressing African horse sickness virus (AHSV) VP2 protects against AHSV challenge in an IFNAR −/− mouse model.. PLoS One.

[31] Potgieter AC, Cloete M, Pretorius PJ, van Dijk AA (2003). A first full outer capsid protein sequence data-set in the Orbivirus genus (family Reoviridae): cloning, sequencing, expression and analysis of a complete set of full-length outer capsid VP2 genes of the nine African horsesickness virus serotypes.. J Gen Virol.

[32] Howell PG, Kumm NA, Botha MJ (1970). The application of improved techniques to the identification of strains of bluetongue virus.. Onderstepoort J Vet Res.

[33] Davies FG, Blackburn NK (1971). The typing of bluetongue virus.. Res Vet Sci.

[34] Blacksell SD, Lunt RA (1996). A simplified fluorescence inhibition test for the serotype determination of Australian bluetongue viruses.. Aust Vet J.

[35] Laegreid WW, Skowronek A, Stone-Marschat M, Burrage T (1993). Characterization of virulence variants of African horsesickness virus.. Virology.

[36] Newsholme SJ (1983). A morphological study of the lesions of African horsesickness.. Onderstepoort J Vet Res.

[37] O'Hara RS, Meyer AJ, Burroughs JN, Pullen L, Martin LA (1998). Development of a mouse model system, coding assignments and identification of the genome segments controlling virulence of African horse sickness virus serotypes 3 and 8.. Arch Virol Suppl.

[38] Jeggo MH, Wardley RC, Brownlie J, Corteyn AH (1986). Serial inoculation of sheep with two bluetongue virus types.. Res Vet Sci.

[39] Maan S, Maan NS, Samuel AR, Rao S, Attoui H (2007). Analysis and phylogenetic comparisons of full-length VP2 genes of the 24 bluetongue virus serotypes.. J Gen Virol.

[40] Maan S, Maan NS, Ross-smith N, Batten CA, Shaw AE (2008). Sequence analysis of bluetongue virus serotype 8 from the Netherlands 2006 and comparison to other European strains.. Virology.

[41] Mertens PPC, Maan NS, Prasad G, Samuel AR, Shaw AE (2007). Design of primers and use of RT-PCR assays for typing European bluetongue virus isolates: differentiation of field and vaccine strains.. J Gen Virol.

[42] Sailleau C, Hamblin C, Paweska JT, Zientara S (2000). Identification and differentiation of the nine African horse sickness virus serotypes by RT-PCR amplification of the serotype-specific genome segment 2.. J Gen Virol.

[43] Potgieter AC, Page NA, Liebenberg J, Wright IM, Landt O (2009). Improved strategies for sequence-independent amplification and sequencing of viral double-stranded RNA genomes.. J Gen Virol.

[44] Mertens PPC, Attoui H (2011). The RNAs and proteins of dsRNA viruses.. http://www.reoviridae.org/dsRNA_virus_proteins/ReoID/AHSV-isolates.htm.

[45] Attoui H, Billoir F, Cantaloube JF, Biagini P, de Micco P (2000). Strategies for the sequence determination of viral dsRNA genomes.. J Virol Methods.

[46] Zientara S, Sailleau C, Moulay S, Cruciere C, el-Harrak M (1998). Use of reverse transcriptase-polymerase chain reaction (RT-PCR) and dot-blot hybridisation for the detection and identification of African horse sickness virus nucleic acids.. Arch Virol Suppl.

[47] Maan S, Maan NS, van Rijn PA, van Gennip RG, Sanders A (2010). Full genome characterisation of bluetongue virus serotype 6 from the Netherlands 2008 and comparison to other field and vaccine strains.. PLoS One.

[48] Maan S, Rao S, Maan NS, Anthony SJ, Attoui H (2007). Rapid cDNA synthesis and sequencing techniques for the genetic study of bluetongue and other dsRNA viruses.. J Virol Methods.

[49] Katoh K, Asimenos G, Toh H (2009). Multiple alignment of DNA sequences with MAFFT.. Methods Mol Biol.

[50] Thompson JD, Higgins DG, Gibson TJ (1994). CLUSTAL W: improving the sensitivity of progressive multiple sequence alignment through sequence weighting, position-specific gap penalties and weight matrix choice.. Nucleic Acids Res.

[51] Tamura K, Dudley J, Nei M, Kumar S (2007). MEGA4: Molecular Evolutionary Genetics Analysis (MEGA) software version 4.0.. Mol Biol Evol.

[52] Vreede FT, Huismans H (1994). Cloning, characterization and expression of the gene that encodes the major neutralization-specific antigen of African horsesickness virus serotype 3.. J Gen Virol.

[53] Iwata H, Yamagawa M, Roy P (1992). Evolutionary relationships among the gnat-transmitted orbiviruses that cause African horse sickness, bluetongue, and epizootic hemorrhagic disease as evidenced by their capsid protein sequences.. Virology.

[54] Koekemoer JJ, Potgieter AC, Paweska JT, Van Dijk AA (2000). Development of probes for typing African horsesickness virus isolates using a complete set of cloned VP2-genes.. J Virol Methods.

[55] Koekemoer JJ (2008). Serotype-specific detection of African horsesickness virus by real-time PCR and the influence of genetic variations.. J Virol Methods.

[56] Koekemoer JJ, Dijk AA (2004). African horsesickness virus serotyping and identification of multiple co-infecting serotypes with a single genome segment 2 RT-PCR amplification and reverse line blot hybridization.. J Virol Methods.

