# Supplementary material for: Serotype Specific Primers and Gel-Based RT-PCR Assays for ‘Typing’ African Horse Sickness Virus: Identification of Strains from Africa
Source: PLoS One. 2011 Oct 20;6(10):e25686. doi: 10.1371/journal.pone.0025686 (PMC3197586; doi:10.1371/journal.pone.0025686)
Supplement: Table S1 — Primers for specific amplification of Seg-2 from various AHSV serotypes in RT-PCR assays. (DOC) [file pone.0025686.s001.doc]

**Table S1:**

| **Primer Pair^1^** | **Primer Name^2,4^** | **Primer Sequence (5’-3’)** | **Position on genome Seg-2 (nt)** | **Predicted Product size (bp)** | **IAH-P^3^ dsRNA virus reference collection of the strain and accession numbers of Seg-2 sequences from which primers were designed** |
| --- | --- | --- | --- | --- | --- |
| 1A1 | AHSV-1/S2/508-532F AHSV-1/S2/2301-2279R | GGTGTGTTGAATGGAAATAAACAG GCTCAATCCTTCACGGTTAAGG | 508-532 2301-2279 | 1793 (Fig. 1 – Panel A) | RSArah1/03, AY163329, FJ011108 |
| 1A2 | AHSV-1/S2/552-570F AHSV-1/S2/2006-1984R | GGAAATGGCGCATCAACGG TTATCTCGAATGTCAGTTCGCTT | 552-570 2006-1984 | 1452 (Fig. 1 – Panel A) | RSArah1/03, AY163329, FJ011108 |
| 2A1 | AHSV-2/S2/456-477F AHSV-2/S2/1554-1535R | RTATCCATTTGATATAAGATG GATGATATCTCCACGGAAG | 456-477 1554-1535 | 1098 (Fig. 1 – Panel A) | RSArah2/03, ETH2010/01, SEN2007/01 - SEN2007/05, AY163332, FJ196585 |
| 2A2 | AHSV-2/S2/577-598F AHSV-2/S2/1916-1892R | GAAGAAAACGACCGTGGAAAR ATGCCCTCAACGTTCTCTGTCTGA | 577-598 1916-1892 | 1339 (Fig. 1 – Panel A) | RSArah2/03, ETH2010/01, SEN2007/01 - SEN2007/05, AY163332, FJ196585 |
| 3A1 | AHSV-3/S2/447-467F AHSV-3/S2/1198-1176R | GGTTTCTCGTTCAATTATAG TYAAAAACTTCTTAACTTCCGCY | 447-467 1198-1176 | 751 (Fig. 1 – Panel A) | RSArah3/03, DQ868772, Z26316, U01832 |
| 3A2 | AHSV-3/S2/493-514F AHSV-3/S2/2017-1998R | GCTGAACAGACGAAGGGAACGA GTATATGTTCGCCACGTGCG | 493-514 2017-1998 | 1524 (Fig. 1 – Panel A) | RSArah3/03, DQ868772, Z26316, U01832 |
| 4A1 | AHSV-4/S2/441-461F AHSV-4/S2/1705-1683R | GCTTGATCGRATTCGGARTT TCACCGCGAAGCCATCCCTACG | 441-461 1705-1683 | 1264 (Fig. 1 – Panel B) | RSArah4/03, KEN2007/01, KEN2007/02, SPA1987/01, M94680, M90697, D26570, U21956, EU046574, DQ868773 |
| 4A2 | AHSV-4/S2/555-576F AHSV-4/S2/2018-1998R | TGCRGTTAATGAAAGGATTRT GGCTTCTGTTTCTCATTCAT | 555-576 2018-1998 | 1463 (Fig. 1 – Panel B) | RSArah4/03, KEN2007/01, KEN2007/02, SPA1987/01, M94680, M90697, D26570, U21956, EU046574, DQ868773 |
| 4A3 | AHSV-4/S2/876-897F AHSV-4/S2/2347-2325R | TTCATCWKCRGGTGATAAGCAG ATCTCARCTYYTTCACATCCTCC | 876-897 2347-2325 | 1471 (data not shown) | RSArah4/03, KEN2007/01, KEN2007/02, SPA1987/01, M94680, M90697, D26570, U21956, EU046574, DQ868773 |
| 5A1 | AHSV-5/S2/437-458F AHSV-5/S2/1589-1567R | TGAATGTTGARATGCTGAGAG GTATTCAGAGTGATTCCAAAGG | 437-458 1589-1567R | 1152 (data not shown) | RSArah5/03, AY163331 |
| 5A2 | AHSV-5/S2/573-599F AHSV-5/S2/1996-1977R | GTCGCGCGAGGGGAATAGTCAAACTATATCAGTTAAAGTCAATAG | 573-599 1996-1977 | 1423 (Fig. 1 – Panel B) | RSArah5/03, AY163331 |
| 5A3 | AHSV-5/S2/714-735F AHSV-5/S2/1853-1828R | CAAAGGAGGTTTGATAGCYAAC TCAAAGACCCTATCTGGCYTRTYAAC | 714-735 1853-1828 | 1139 (Fig. 1 – Panel B) | RSArah5/03, AY163331 |
| 6A1 | AHSV-6/S2/473-496F AHSV-6/S2/1627-1606R | GRATGCGARRGATTGARGCYAGG GCATCGTCGYTCRACGAATAR | 473-496 16271606 | 1154 (Fig. 1 – Panel C) | RSArah6/03, ETH2010/19, NC_005996, AF021235 |
| 6A2 | AHSV-6/S2/501-521F AHSV-6/S2/1771-1748R | RCGAATTARGGAGGGYGRTG GCTCTTTCTTCCTAAGTTTYGTG | 501-521 1771-1748 | 1270 (Fig. 1 – Panel C) | RSArah6/03, ETH2010/19, NC_005996, AF021235 |
| 6A3 | AHSV-6/S2/693-715F AHSV-6/S2/1972-1948R | TCCRACARCBGTGACTAGAACTT GCTTCGTTGTTATTTTCTCAACACG | 693-715 1972-1948 | 1298 (data not shown) | RSArah6/03, ETH2010/19, NC_005996, AF021235 |
| 7A1 | AHSV-7/S2/491-513F AHSV-7/S2/1917-1894R | GTARCGCACAGACGAAAYACGT GCGTTGYCCCTCTCGAAGTGTAG | 491-513 1917-1894 | 1426 (Fig. 1 – Panel C) | KENrah7/03, SEN2007/06, ETH2010/09, AY163330 |
| 7A2 | AHSV-7/S2/573-593F AHSV-7S2/1999-1978R | GATGGAGGGCCAACAAGAGA GYATATCGAYTCTCGWCATCG | 573-593 1999-1978 | 1426 (Fig. 1 – Panel C) | KENrah7/03, ETH2010/09, AY163330 |
| 8A1 | AHSV-8/S2/586-609F AHSV-8/S2/2343-2321R | GTGGGAAAGARAGTGTGTGTAAG GTCTTTCTTAGTYAGTCCGCTG | 586-609 2343-2321 | 1757 (Fig. 1 – Panel D) | RSArah8/03, ETH2010/10, ETH2010/11, AY163333, DQ868775 |
| 8A2 | AHSV-8/S2/569-589F AHSV-8/S2/2301-2274R | GTATGTTAGAGGAGGGAGTG ATGTGCTGAATCCACYCTATACCTCYGG | 569-589 2301-2274 | 1732 (Fig. 1 – Panel D) | RSArah8/03, ETH2010/10, ETH2010/11, AY163333, DQ868775 |
| 9A1 | AHSV-9/S2/487-509F AHSV-9/S2/1970-1947R | GAGCGAGARTTARGKRSTGGRG GARGTBTGAACCTGTCGAACTCG | 487-509 1970-1947 | 1483 (Fig. 1 – Panel D) | PAKrah9/03, ETH2010/23, SENvvvv/09, AF043926, DQ868776 |
| 9A2 | AHSV-9/S2/585-606F AHSV-9/S2/2291-2272R | GACGATGTGYGTGAGRCATGA GAAAGATCAGYYTCCTCAG | 585-606 2291-2272 | 1706 (Fig. 1 – Panel D) | PAKrah9/03, ETH2010/23, SENvvvv/09, AF043926, DQ868776 |

^1^ Primer-pairs identified by serotype, the letter A, indicating ‘AHSV’ were selected to detect all available isolates of each AHSV serotype [44] and the number of primer- pair.

^2^ Individual primers are identified by the AHSV serotype (e.g. AHSV-1) followed by the S2 (to indicate Seg-2), then a range of numbers to indicate the relative start and end nucleotide position of the primer within segment 2, followed by F or R to indicate forward or reverse orientation respectively.

^3^ Institute for Animal Health, Pirbright.

^4^ All primer-pairs were used as described previously by Maan et al [47], with annealing conditions - 55^0^C for 30 seconds.
